# Glances at the Hospitals

**Published:** 1903-07-04

**Authors:** 


### GLANCES AT THE HOSPITALS.

### GREAT YARMOUTH HOSPITAL.

THE total ordinary income was £2,501, and the total ordinary expenditure was £2,535. The extraordinary income was £1,333, and the extraordinary expenditure was £100. The number of in-patients was 467, the daily average of beds occupied was 45, and the average duration of each patient's stay in the hospital was 35 days. The latter seems very high, although it may be susceptible of satisfactory explanation. Of the total number under treatment, 247 were cured, 141 were relieved, 32 died, and 47 remained under treatment. Eight of the deaths took place within 24 hours of admission. The medical and surgical tables are prepared with columns showing total numbers, sex of patients, cured, died, and remaining under treatment, hence we can get the results without difficulty. Of acute pneumonia there were 9 cases, with 1 death—an alcoholic. There were 6 cases of consumption, and of these 4 were cured and 2 died. Of 16 cases of abdominal section, there were 13 recoveries and 3 deaths. The total number of major operations was 59, of minor operations 265. Ether was administered 144 times, chloroform 80, chloroform and ether 15, A.C.E. mixture 22, nitrous oxide 52, and cocaine 10. The total number of out-patients was 2,567.

### ROYAL EYE HOSPITAL, SOUTHWARK, S.E.

THE total ordinary income amounted to £2,172, and the total ordinary expenditure to £3,319, showing a deficit of £1,147; but the extraordinary income practically covered this deficit. The Governors point out that to keep open all the beds and to provide for the increase in the out-patients' department an income of £4,500 is necessary; and when it is stated that 19,770 sufferers, of whom 629 were in-patients, were relieved last year, it can hardly be said that the Governors are placing their wants at a high figure. The average stay in the hospital was 16 days; and the cost of each in-patient was £2 9s., and of each out-patient 1s. 10½d. During the year 557 operations were performed. In 163 of these a general anæsthetic was administered, and in the others cocaine or cocaine with adrenalin was employed. It is most satisfactory to find that only one eye had to be removed after operation, and this was owing to intra-ocular hæmorrhage following cataract extraction There were 101 operations for cataract, and of these 95 were wholly successful, 5 partially successful, and 1 failed.

### TUNBRIDGE WELLS GENERAL HOSPITAL.

THE total ordinary income was £3,297, and the ordinary expenditure was £3,467. The hospital is being enlarged at a cost of £21,983. As the old hospital only contained 55 beds, it is clear that the new buildings and the alterations must be of an extensive nature. At the beginning of the year there were 44 patients in the hospital and 525 were admitted during the year making a total of 569 under treatment. Of these 421 were discharged recovered, 64 relieved, 8 not relieved, 40 died, and 36 remained under treatment. The out-patients numbered 4,326. We fail to notice any statement of the cost per patient, or of the cost per bed occupied. The medical and surgical reports are contained on a single page, and give us no information beyond the mere numbers suffering from the diseases named. There were 296 operations, but no results are given, nor is there any table showing the nature of the anæsthetics used.

### FOSTER-GREEN HOSPITAL FOR CONSUMPTION, BELFAST.

THE income amounted to £1,982 and the expenditure to £2,318, showing a deficit of £336. Fortunately the hospital has a good friend in Mr. Foster-Green, and he has offered £150 on condition that the public will clear off the remainder of the debt; and there cannot be a doubt that this sum will be immediately raised, if it has not been already subscribed previous to the report falling into our hands. The value of the open-air treatment has been fully demonstrated at many sanatoria, both on the Continent and in England, and the experience of every hospital is conclusive in its favour, so that the public need not hesitate to support these institutions. There were 33 patients in this hospital on January 1st, and 163 were admitted during the year. On admission the average evening temperature was 99·8°, and on discharge it was 98·8°. The average evening pulse on admission was 106, and on discharge it was 98. The condition of the patients on admission is summed up as follows:—slightly affected 15, seriously affected 29, moderately affected 62, very seriously affected 56. On discharge we find that in 12 the disease was arrested, in 75 the condition was much improved, in 53 there was improvement and in 22 no improvement. It would be interesting to know how many of the 12

cases arrested came from the 15 cases returned as slightly affected. On looking over the table showing the previous occupations of the patients, we find that 13 were clerks, 17 housewives, 6 of no occupation, 4 were shop assistants, 4 were weavers, 3 were carpenters, 5 were servants, and 3 were farmers. No other occupation had more than 2 cases belonging to it. Of course no very definite conclusion can be drawn from the above figures unless we knew the proportion which the healthy members of the above-named professions bear to the others, but the fact that out-of-door workers suffer less than others has long been known.

